# Hyponatremia during hospitalization and in-hospital mortality in patients hospitalized from heart failure

**DOI:** 10.1186/s12872-015-0082-5

**Published:** 2015-08-14

**Authors:** S. Saepudin, Patrick A. Ball, Hana Morrissey

**Affiliations:** School of Psychological and Clinical Sciences, Charles Darwin University, Northern Territory, Australia; Department of Pharmacy, Universitas Islam Indonesia, Yogyakarta, Indonesia

## Abstract

**Background:**

To date, the majority of studies on hyponatremia focussed on hyponatremia at admission, and came from developed countries. This study aimed to identify the prevalence of hyponatremia during hospitalization in patients hospitalized for HF and its association with in-hospital mortality.

**Methods:**

This was an observational study using retrospective data from patients’ records between 2010–2013. It focused on those patients carrying an ICD-10 code of 150.0(Congestive Heart Failure) as their primary diagnosis.

Hyponatremia during hospitalization was defined as serum sodium level lower than 135 mEq/L obtained from a blood chemistry measurement on the next days after admission. Patients’ characteristics were examined and the association between hyponatremia during hospitalization and in-hospital mortality was analyzed.

**Results:**

Among 464 patients hospitalized for HF, hyponatremia during hospitalization was observed in 22 % of patients with 44 % of this group had normal serum sodium level on admission.

Hyponatremia during hospitalization was associated with lower blood pressure on admission, both systolic and diastolic, peripheral oedema, ascites and fatigue. Patients having history of hospitalization for cardiac diseases and renal failure were higher in patients developing hyponatremia during hospitalization. In this group, amiodarone, heparin, insulin and antibiotics were administered more frequently. Factors potentially increase the risk of hyponatremia during hospitalization include history of fatigue (OR = 3.23, 95 % CI 1.79–5.82), presence of ascites (4.14, 1.84–9.31), and administration of heparin (3.85, 1.78–8.31) and antibiotics (3.08, 1.71–5.53). Length of hospital stay was significantly longer in patients with hyponatremia during hospitalization and in-hospital mortality was also higher compared to non-hyponatremic patients, 7.7 % and 29.1 %, respectively.

**Conclusion:**

This study found that the prevalence of hyponatremia during hospitalization in patients hospitalized for HF was almost the same as hyponatremia on admission and administration of heparin and antibiotics can potentially worsen hyponatremia during hospitalization. In this study population, hyponatremia during hospitalization was found to be associated with in-hospital mortality.

## Background

Hyponatremia is an under-rated problem in managing patients with heart failure (HF). It shares many pathophysiologic and prognostic features with HF [1, 2]. Patients with HF have a high probability of suffering from hyponatremia, either as a result of disease progression or the effects of medications [3, 4]. Diuretics cause fluid loss by excreting sodium, and medications that inhibit the production or action of aldosterone, (angiotensin converting enzyme inhibitors, angiotensin receptor blockers, spironolactone) prevent sodium re-uptake from the kidney. Additionally hyponatremia is a strong independent predictor of quality of life and mortality in patients with HF [5–11]. In both hospitalized patients, and those in the community, the role of sodium depletion as a predictor of short-term and long-term prognosis, has been documented [12, 13].

In HF patients, hyponatremia may occur through a complex process of pathophysiology related to the changes contributing to HF, including hormonal and neurological disorders [3, 14]. Chronic activation of the rennin-angiotensin-aldosterone system (RAAS) concurrently with stimulation of the sympathetic nervous system as a response to inadequate tissue perfusion, stimulates a counter-productive effect including cardiac remodeling and water and sodium retention [2, 3]. Arginine-vasopressin (AVP) is also released as a response to low cardiac output, to increase intravascular volume. However, the effect is further counter-productive for cardiac workload as preload is increased [1, 15, 16].

The risk of hyponatremia among patients with HF is associated with the severity of the HF [11]. When ventricular dysfunction is severe, the counter-productive neurohormonal response will also increase, leading to excessive water reabsorption, after which hyponatremia can occur [15]. The lower the cardiac output, the greater the AVP release. Prolonged elevation of this hormone in the systemic circulation, results in an increase of water retention leading to a dilution process that may result in hyponatremia [3, 4].

Most published studies on hyponatremia in patients with HF have been conducted in developed countries with advanced resources. Additionally, most studies focused on the prevalence of hyponatremia on admission and its association with in-hospital mortality or long-term prognosis. Studies on hyponatremia from developing countries with limited resources, and also studies focusing on hyponatremia during hospitalization are still lacking. This study aimed to assess the prevalence of hyponatremia during hospitalization in patients hospitalized from HF and its relationship with in-hospital mortality.

## Methods

This was an observational retrospective study conducted at Fatmawati Hospital, a tertiary teaching hospital, located in Jakarta Indonesia. A cross-sectional study was designed to assess the prevalence of hyponatremia during hospitalization and its relationship with in-hospital mortality. Patients hospitalized for HF between January 2010 and December 2013 aged 18 years or older, coded with I50.0 according to International classification of diseases, 10th edition (ICD-10) system and having a reasonably complete record during hospitalization were included in this study. Patients diagnosed as having any malignancy, hepatic cirrhosis, pregnant women and patients on dialysis, and those with missing records were excluded from this study. Patient information collected included demographic profiles, vital signs and symptoms at admission, past medical history, medication during hospitalization and blood chemistry profiles. All were retrieved manually from medical records.

In this study, a patient was categorized as hyponatremic if serum sodium level was lower than 135 mEq/L [8, 9]. A patient was categorized as developing hyponatremia during hospitalization, if at least one episode of hyponatremia occurred on the next day after admission regardless of serum sodium level on admission. Serum sodium levels were corrected for patients with blood glucose level >200 mg/dL using correction factor of 2.4 per 100 mg/dL increase of blood glucose level [17]. In–hospital mortality in this study was defined as death from any cause during hospitalization.

Continuously variable data with normal distribution are presented as mean ± SD, and Student’s t-test was used to compare the means of the groups. If the data was not normally distributed, median with interquartile ranges is quoted and Mann–Whitney U tests were used to compare groups, respectively. Nominal data were presented as proportion (percentage) and Chi-square tests were used to compare the groups. Logistic regression analysis was performed to identify variables associated with hyponatremia during hospitalization by including all variables available at admission and during hospitalization as independent variables. Logistic regression analysis was also performed to assess the relationship between hyponatremia, both at admission and during hospitalization, and in-hospital mortality. Two tailed p values < 0.05 and odd ratios were considered to indicate a statistically significant relationship. All statistical analyses were performed using Statistical Package for Social Sciences software for Windows version 22.0 (SPSS Inc., Chicago, USA).

This study was conducted in accordance with the regulations on extracting patients’ information from medical records established by Ministry of Health Republic of Indonesia and has been approved by Fatmawati Hospital Ethics Committee and The Charles Darwin University Ethics Committee.

## Results

During 2011–2013, 543 hospitalized patients were coded with I50.0 for their main diagnosis of hospitalization. Seventy-nine patients were excluded, due to incomplete laboratory records, pregnancy or routine hemodialysis. Compared to other electrolyte disturbances, this study found that hyponatremia, both on admission and during hospitalization, was the most prevalent. Table 1 shows that the prevalence of hyponatremia in patients hospitalized for HF is around double that for hypokalemia.

**Table 1:** Comparison between sodium and potassium disturbances observed in patients hospitalized for HF

Type of electrolyte abnormalities	Prevalence based on time of occurrence	
	On admission	During hospitalization
Hyponatremia	19 %	22 %
Hypernatremia	<1 %	1 %
Hypokalemia	10 %	11 %
Hyperkalemia	7 %	4 %

**Fig. 1:**
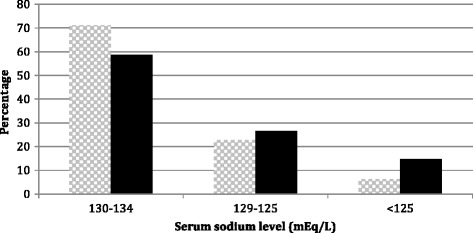
Distribution of serum sodium level in patients with hyponatremia.  on admission and  during hospitalisation

In 464 hospitalized patients with HF included in this study, hyponatremia was found in 19 % on admission and 22 % during hospitalization. Distribution of serum sodium levels is shown in Fig. 1. The mean lowest measured serum sodium level in patients with hyponatremia during hospitalization was not significantly different from the mean lowest serum sodium level of patients with hyponatremia on admission, 129.8 ± 6.2 mEq/L and 130 ± 4.2 mmEq/L, respectively, but hyponatremia during hospitalization comprised more severe serum sodium depletion (p < 0.001).

As this study focused on hyponatremia during hospitalization, comparison of patients’ characteristics was listed between patients with and without hyponatremia during hospitalization as shown in Table 2. Compared to those with normonatremia, patients with hyponatremia during hospitalization were of similar age and gender. However patients with hyponatremia had lower blood pressure, both systolic and diastolic, more peripheral oedema, ascites and fatigue; but chest pain was found to be less frequent. In term of past medical history, the proportion of patients having a history of hypertension was lower in patients with hyponatremia but the proportion of patients having a history of hospitalization for cardiac diseases was higher. Among concomitant diagnoses, renal failure was found more prevalence in hyponatremic patients. Other than the xserum sodium and chloride levels, patients with hyponatremia during hospitalization had significantly different levels of blood urea nitrogen, lipid profile, albumin and hepatic enzymes levels (Alanine transaminase– ALT and Aspartate transaminase- AST) from patients without hyponatremia. Hyponatremic patients had lower total cholesterol and high density lipoprotein (HDL) levels. Serum albumin of patients with hyponatremia during hospitalization was lower than patients without, and this might be associated with the higher proportion of patients having ascites in the hyponatremic group. Elevated hepatic enzymes level, both AST and ALT, were more prevalent in patients with hyponatremia during hospitalization.

**Table 2:** Comparisons of patients’ characteristics between patients with normonatremia and patients with hyponatremia during hospitalization

Variable	Non-hyponatremia during hospitalisation (*n* = 362)	Hyponatremia during hospitalization (*n* = 102)	P Value
Age (yrs)	54.2 ± 15.3	52.1 ± 12.9	0.162
Sex (Male)	53.6 %	50.9 %	0.641
Ejection fraction (%) *	39.3 ± 18.4	39.9 ± 20.1	0.846
Ischemia as cause of HF	58.3 %	51.9 %	0.254
Vital signs and symptoms on admission			
SBP	129.4 ± 27.4	117.8 ± 24.1	<0.001
DBP	83.1 ± 18.2	76.9 ± 16.8	0.002
Heart rate	94.8 ± 17.6	97.4 ± 18.9	0.212
Respiratory rate	26.7 ± 5.9	27.6 ± 6.1	0.181
Orthopnea	69.6 %	71.6 %	0.703
PND	52.5 %	58.8 %	0.257
Cough	18.8 %	18.6 %	0.971
Chest pain	20.7 %	7.8 %	0.003
Peripheral oedema	61.1 %	72.5 %	0.033
Ascites	8.8 %	21.6 %	<0.001
Fatigue	21.5 %	50.9 %	<0.001
Medical history			
Hypertension	45.1 %	31.4 %	0.014
Diabetes mellitus	22.7 %	24.5 %	0.694
Chronic obstructive pulmonary disease	4.1 %	7.8 %	0.128
Stroke	3.9 %	1.9 %	0.351
Tuberculosis	4.7 %	7.8 %	0.214
Asthma	2.2 %	2.9 %	0.509
Previous hospitalization for cardiac disease	40.9 %	51.9 %	0.046
Concomitant diagnosis			
Atrial fibrillation	15.5 %	12.7 %	0.495
Pneumonia	15.7 %	20.6 %	0.248
Renal failure	17.7 %	33.3 %	0.001
Cardiac arrhythmia	4.9 %	5.9 %	0.714
Infectious diseases (other than pneumonia)	4.1 %	5.9 %	0.456
Blood chemistry at admission			
Sodium (mEq/L)	139.9 ± 4.5	133.1 ± 6.2	<0.001
Potassium (mEq/L)	4.4 ± 0.4	4.2 ± 0.9	0.824
Chloride (mEq/L)	102. 9 ± 7.4	96.2 ± 8.1	<0.001
Blood glucose (mg/dL)	142.1 ± 73.2	137.1 ± 76.7	0.555
Blood urea nitrogen (mg/dL)	50.7 ± 33.7	73.1 ± 50.9	<0.001
Creatinine (mg/dL)	1.4 ± 1.1	1.7 ± 1.4	0.075
Uric acid (mg/dL)	9.4 ± 5.7	9.0 ± 4.0	0.633
Total cholesterol (mg/dL)	158.0 ± 48.3	137.9 ± 43.1	<0.001
HDL (mg/dL)	35.5 ± 17.9	27.4 ± 12.6	<0.001
Protein total (g/dL)	6.5 ± 0.9	6.3 ± 1.2	0.097
Albumin (g/dL)	3.4 ± 0.6	3.2 ± 0.5	<0.001
Hemoglobin (g/dL)	12.7 ± 2.5	12.8 ± 2.3	0.919
Erythrocyte (x10^12^/L)	4.61 ± 2.16	4.61 ± 1.23	0.996
Hematocrit (%)	39.5 ± 7.0	40.2 ± 6.9	0.544
Leucocyte (x10^9^/L)	8.3 (6.7–10.5)	8.7 (7.0–12.3)	0.187
AST (IU/L)	34 (25–55)	40 (28–83)	0.012
ALT (IU/L)	26 (16–51)	34 (18–88)	0.011

Table 3 listed medication administered to patients hospitalized for HF during hospitalization. While the proportion of patients receiving diuretics, both furosemide and potassium sparing diuretics, was not different, the proportion of patients receiving angiotensin converting enzyme inhibitors (ACEIs) or angiotensin receptor blockers (ARBs) was lower in patients with hyponatremia during hospitalisation. The number of patients receiving amiodarone, heparin, insulin and antibiotics was higher in those developing hyponatremia during hospitalization compared to others without hyponatremia.

**Table 3:** Medication administered during hospitalization

Medication	Non-hyponatremia during hospitalization (*n* = 362)	Hyponatremia during hospitalization (*n* = 102)	P value
Furosemide	95.3 %	95.1 %	0.931
ACEIs or ARBs	77.3 %	64.7 %	0.01
Sparing diuretics	24.3 %	29.4 %	0.296
Amiodarone	15.5 %	22.5 %	0.093
Potassium supplement	55.5 %	53.9 %	0.774
Positive inotropes	9.1 %	31.4 %	<0.001
Organic nitrates	62.2 %	54.9 %	0.186
Digoxin	25.1 %	26.5 %	0.785
Aspirin	48.1 %	43.1 %	0.378
Clopidogrel	28.7 %	33.3 %	0.369
Simvastatin	46.4 %	41.2 %	0.348
Heparin	9.1 %	18.6 %	0.007
Warfarin	26.5 %	21.6 %	0.311
CCBs	14.4 %	8.8 %	0.144
Ubiquinone	14.7 %	10.8 %	0.314
Beta blockers	13.5 %	9.8 %	0.318
Laxative agents	40.9 %	38.2 %	0.630
PPIs	14.9 %	22.5 %	0.069
H_2_RAs	22.1 %	30.4 %	0.083
Allopurinol	18.8 %	20.6 %	0.683
Benzodiazepine	21.8 %	22.5 %	0.876
Insulin	7.5 %	13.7 %	0.049
Oral anti-diabetics	6.6 %	5.9 %	0.786
Antibiotics	35.4 %	71.6 %	<0.001
Beta blockers on discharge	10.8 %	12.9 %	0.555
Length of hospital stay	8 (5–12)	11 (8–15)	<0.001
In-hospital death	7.7 %	29.1 %	<0.001

Table 4 lists variables associated with hyponatremia during hospitalization found during multivariable logistic regression analysis. Other than serum sodium level at admission, other factors associated with hyponatremia during hospitalization found in this research are history of fatigue, history of hypertension, presence of ascites at admission, and administration of heparin and antibiotics during hospitalization.

**Table 4:** Factors associated with hyponatremia during hospitalization

Variable	P value	Odds ratio	95 % Confidence interval
History of fatigue	<0.001	3.23	1.79–5.83
History of hypertension	0.022	0.49	0.27–0.91
Serum sodium at admission	<0.001	0.77	0.73–0.83
Presence of ascites	0.001	4.14	1.85–9.31
Administration of heparin	0.001	3.85	1.78–8.31
Administration of antibiotics	<0.001	3.08	1.71–5.53

Patients developing hyponatremia during hospitalization showed a significantly longer length of hospital stay compared to patients without hyponatremia, with median and interquartile range at 11 (8–15) and 8 (5–12) days, respectively. In-hospital mortality rate was also observed higher (p < 0.001) in hyponatremic patients compared to patients without hyponatremia, at 29.1 % and 7.7 %, respectively.

In term of in-hospital mortality, as showed in Table 5, while hyponatremia on admission has no association with in-hospital mortality, hyponatremia during hospitalization has an association with odds ratio 3.473 (95 % CI 1.899–6.351).

**Table 5:** Association between hyponatremia and in-hospital mortality

Type of hyponatremia based on time of occurrence	P value	Odds ratio	95 % CI
Hyponatremia on admission	0.054	1.874	0.989–3.551
Hyponatremia during hospitalization	<0.001	3.473	1.899–6.351

## Discussion

The present study clearly demonstrates that hyponatremia is the most prevalent electrolyte disturbance in patients hospitalized for HF [7, 8, 10]. Based on information on vital signs and symptoms at admission, this study also shows that patients with hyponatremia presented with more severe disease and their prognosis, in term of in-hospital mortality, was also worse. Compared to patients without hyponatremia during hospitalization, peripheral edema and ascites at admission were found to be more prevalent in patients who developed hyponatremia during hospitalization. Patients with more severe HF would potentially have these symptoms as a result of poor cardiac function, i.e. more severe ventricular dysfunction. Left ventricular ejection fraction (LVEF) has been well known as an indicator of cardiac pump function, in which lower LVEF indicates poorer cardiac pump function [12]. In this study, the average of LVEF is not significantly different between patients with normonatremia and patients with hyponatremia during hospitalization, but the average was calculated from only 47.6 % and 43.4 % of patients with and without hyponatremia during hospitalization, respectively. In their research, Sato et al. reported signs of a difference LVEF between these groups but this was not significant [8].

This study found that a higher proportion of patients developing hyponatremia during hospitalization had a history of hospitalization for cardiac diseases but the disease was not mentioned specifically in medical records. Previous published studies also reported that more hyponatremic patients have previous hopstalization for HF compared to nonhyponatremic patients [8, 13] and this might be related to the poorer condition of HF in hyponatremic patients. Renal failure was the only one concomitant diagnosis found in this study with a significant correlation between hyponatremic and non-hyponatremic patients but serum creatinine of both groups was found not significantly different. However, the average of blood urea nitrogen in hyponatremic patients was found higher than non-hyponatremic patients. In acute conditions, serum creatinine of HF patients may be increased owing to hypoperfusion and congestion [18] and worsening renal function in HF patients with congestion has been found as a predictor for poorer prognosis [19]. Liver function abnormality, detected by AST and ALT, was also found higher in patients with hyponatremia during hospitalization in this study. In HF patients, liver function abnormalities indicate the presence of cardio-hepatic syndrome and, specifically, higher level of AST and or ALT indicates ischemia within hepatocytes that should be considered both in managing the patients and predicting of long-term outcome [20].

In term of medication administered during hospitalization, patients with hyponatremia during hospitalization received less ACEIs or ARBs compared to patients with normonatremia. In contrast, patients with hyponatremia received more amiodarone, heparin, insulin and antibiotics. This information on medication might also indicate more severe condition of patients with hyponatremia. The most used ACEIs or ARBs are administered orally and patients with severe condition would have difficulty to take oral medication. In contrast, amiodarone and heparin are administered parenterally and mostly used in patients with severe conditions. However, the rate of overall use of ACEIs or ARBs found in this study at 75 % is higher compared to the rate reported by Callender et al. [21] in their systematic review on HF in low-middle income countries and Siswanto et al. [22] in their study on HF in Indonesia, 57 % and 68 %, respectively.

Other than patient’s condition before and at admission, this study found that medication administered during hospitalization could also worsen hyponatremia. While higher serum sodium level at admission and history of hypertension lowers the risk of hyponatremia during hospitalization, history of fatigue before admission and the presence of ascites at admission conversely increase the risk. Patients received heparin and antibiotics in this study appeared to have around a three fold higher risk of developing hyponatremia during hospitalization with odds ratio 3.85 (95 % CI 1.78-8.31) and 3.08 (1.71-5.53), respectively. While heparin has been known can induce hyponatremia [23], administration of antibiotics might induce hyponatremia by involving a complex association with pathophysiological process of infection.

The overall in-hospital mortality rate found in this study is 11 % and this is higher than the average of in-hospital mortality rates reported by Siswanto, et al. in their report on behalf of The Acute Decompensated Heart Failure National Registry (ADHERE) research team in Indonesia at 6.7 % [22].

This is also higher compared to in-hospital mortality rates of HF patients in developing countries reported by Callender, et al. [21] and in Asia Pacific reported by Atherton, et al. [24], 8 % and 4.8 %, respectively. The higher in-hospital mortality rate found in this study might be due to more severe conditions of the patients included in this study. In their report, Siswanto, et al. found that patients hospitalized for HF in Indonesia tend to have severe symptoms and lower LVEF [22]. It is a challenge for primary care providers and general practitioners in Indonesia to improve management of HF so that patients with HF will be not delayed to receive appropriate treatment [25].

While previous studies have revealed the association between hyponatremia on admission and in-hospital mortality in patients hospitalized for HF [8, 26, 27], the results of this study are slightly different. Instead of hyponatremia on admission, hyponatremia during hospitalization was found to have an association with in-hospital mortality as shown in Table 4. In this study, only 56 % of patients developing hyponatremia during hospitalization were hyponatremic on admission and this means that 45 out of 464 patients (9.7 %) included in this study developed hospital-acquired hyponatremia. In a study with unselected patients, hospital-acquired hyponatremia was found in around one third of hospitalized patients and the condition was associated with increase of length of hospital stay and in-hospital mortality [28]. Therefore, factors associated with increased risk of developing hyponatremia during hospitalization in patients hospitalized for HF are important to be studied.

Other than serum sodium levels several other factors should be considered to assess the hyponatremic status of a patient hospitalized for HF. Although HF patients have a high probability of developing hypervolemic hyponatremia, the possibility of the occurrence of pseudo-hyponatremia and other types of hyponatremia need also to be considered in order to administer appropriate management. Pseudo-hyponatremia, for instance, should be considered in HF patients with hyperglycemia or hypercholesterolemia [29].

As the use of arginine vasopressin receptor antagonists, also known as the vaptans, in patients with HF have been approved, American College Cardiology Foundation/American Heart Association put these drugs on their recommendation for managing HF patients developing hypervolemic hyponatremia [30]. However, role of the vaptans in reducing all-cause mortality and cardiovascular mortality in patients with HF, including their acceptability for long-term use, are still questionable [31–33].

While the vaptans are now might be available in some developed countries, it is not easy to provide these drugs in developing countries due to the cost of the medication. Therefore, the first strategy to minimize hyponatremia-related problem in patients hospitalized for HF should be to optimize guideline-driven therapy and to assess hyponatremia more appropriately [34–37]. Furthermore, conventional options for managing hyponatremia such as the use of saline solution, either isotonic or hypertonic, are still important to be considered [29, 38, 39].

### Limitations

This study was conducted in a single tertiary referral hospital, as the number of tertiary hospitals in Indonesia are more limited compared to secondary hospitals, this study might not be true representation of the whole population hospitalized for HF In Indonesia. Hence, further studies involving more centers and secondary hospitals need to be conducted to get better picture on hyponatremia in patients hospitalized for HF in Indonesia.

Patients included in this study were only HF patients hospitalized with code I50.0 as their main diagnosis in which the code is only for patients with congestive HF and patients with right ventricular failure (secondary to left HF). Therefore, other types of HF were not included in this study.

Furthermore, hyponatremia in this study was only assessed by serum sodium level. Hence, patients’ hyponatremic status could not be differentiate whether it was euvolemic, hypervolemic or might be pseudo-hyponatremia.

As frequently occurrs with retrospective studies, some important information, such as information on medication history before hospitalization, could not also be gathered in this study.

## Conclusion

This study confirmed the findings of previous studies on hyponatremia in patients hospitalized for HF in which hyponatremia was reported as the most prevalent electrolyte disturbances. In addition, this study found that 56 % of patients having hyponatremia on admission continue to be hyponatremic during hospitalization. Factors increasing the risk of hyponatremia during hospitalization found in this study include history of fatigue, the presence of ascites, and administration of heparin and antibiotics. In this study population, in-hospital mortality was found to be more associated with hyponatremia during hospitalization than hyponatremia on admission.
